# Formation and Function of Mammalian Epithelia: Roles for Mechanosensitive PIEZO1 Ion Channels

**DOI:** 10.3389/fcell.2019.00260

**Published:** 2019-10-31

**Authors:** Teneale A. Stewart, Felicity M. Davis

**Affiliations:** ^1^Faculty of Medicine, Mater Research-The University of Queensland, Brisbane, QLD, Australia; ^2^Translational Research Institute, Brisbane, QLD, Australia

**Keywords:** mechanotransduction, PIEZO1, calcium channel, calcium signaling, epithelial biology

## Abstract

Mechanical forces play important roles in shaping mammalian development. In the embryo, cells experience force both during the formation of the mammalian body plan and in the ensuing phase of organogenesis. Physical forces – including fluid flow, compression, radial pressure, contraction, and osmotic pressure – continue to play central roles as organs mature, function, and ultimately dysfunction. Multiple mechanisms exist to receive, transduce, and transmit mechanical forces in mammalian epithelial tissues and to integrate these cues, which can both fluctuate and coincide, with local and systemic chemical signals. Drawing near a decade since the discovery of the bona fide mechanically activated ion channel, PIEZO1, we discuss in this mini-review established and emerging roles for this protein in the form and function of mammalian epithelia.

## Introduction

The transformation of a fertilized egg into a multicellular organism in the reproductive tract of female mammals is a complex and multifactorial process that is completed over a period of less than 3 weeks in mice. Visualization of this process (from gastrulation to early organogenesis) *in toto* at single cell resolution has recently been achieved using adaptive light-sheet microscopy (McDole et al., 2018), producing a dynamic map of embryogenesis and the cellular rearrangements that occur as mammalian organs form and take shape. These data reinforce pioneering studies in vertebrate models describing force production and propagation during gastrulation and organogenesis (Lewis, 1947; Beloussov, 1980; Keller et al., 2003; Krieg et al., 2008) and provide compelling visual evidence that mechanisms-of-multicellularity and mechanosensing in mammals are inextricably linked (Nelson et al., 2005; Orr et al., 2006; Miller and Davidson, 2013). The shaping of epithelial tissues is no exception (Behrndt et al., 2012; Guo et al., 2012).

**FIGURE 1:**
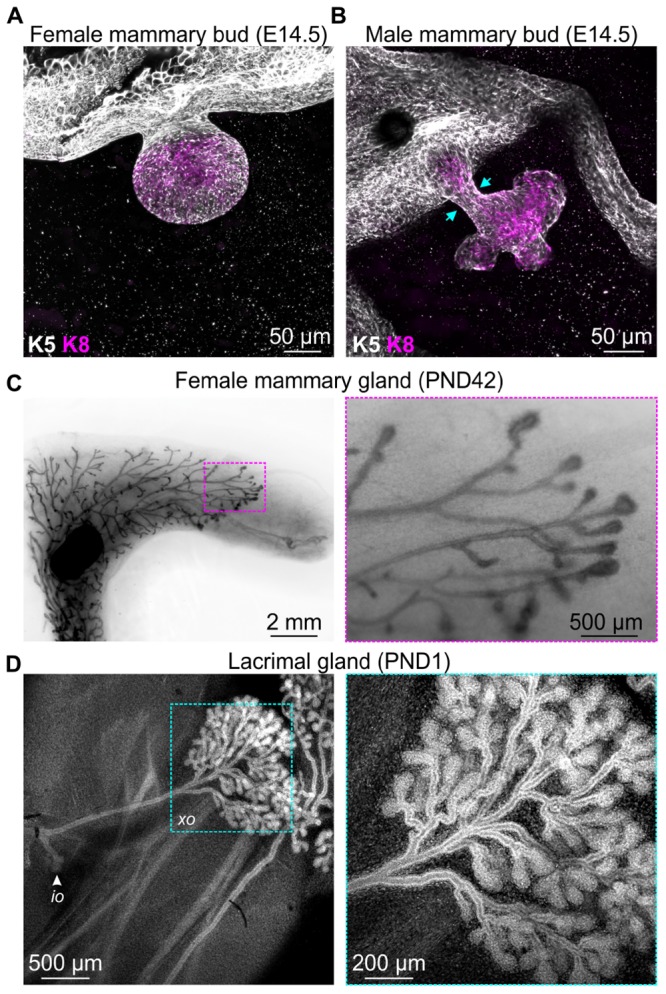
Examples of events that occur in mouse epithelial tissues during development that are associated with extensive cellular rearrangements and morphological changes. 3D confocal imaging of **(A)** female and **(B)** male mouse mammary buds at embryonic day E14.5 immunostained with epithelial cell markers keratin K8 (magenta) and K5 (gray). **(C)** Mammary gland wholemount (methyl green) shows pubertal epithelial branching in the female mouse at postnatal day (PND) 42. Boxed region shows terminal end buds, enlarged in the adjacent sub-panel. **(D)** 3D confocal imaging of mouse lacrimal gland branching at PND1. Cells are stained with DAPI (gray). Arrow indicates the intra-orbital lobe (*io*). Boxed region shows the exorbital lobe (*xo*) and is enlarged in the adjacent sub-panel.

Development of the mammary gland, the namesake epithelial organ of mammals, commences at around E10 in mice with the formation of the mammary lines, which resolve into five pairs of placodes by E11.5 (Cowin and Wysolmerski, 2010). Expansion of the placodes cause these structures to physically bulge above the plane of the surrounding epidermis and ultimately to invaginate into the underlying mesenchyme, creating a series of light bulb-shaped (ball and stick) mammary buds (Figure 1A; Watson and Khaled, 2008; Cowin and Wysolmerski, 2010). This early reorganization of mammary stem cells and their progeny also corresponds with the elongation, condensation, and radial rearrangement of surrounding cells to create the primary mesenchyme (Cowin and Wysolmerski, 2010).

Of particular interest in the context of mechanobiology, the mesenchyme surrounding the epithelial stalk of male mice condenses to such a degree that it physically amputates the mammary bud from the overlying epidermis (Figure 1B; Dunbar et al., 1999; Heuberger et al., 2006), a process that presumably generates considerable compressive forces on epithelial cells. The intact female bud, however, later sprouts to invade the embryonic fat pad, with subsequent rounds of branching morphogenesis occurring both prenatally in the late embryo and postnatally at puberty (Figure 1C; Watson and Khaled, 2008). Similar branching morphogenesis is observed in other epithelial tissues including lacrimal gland (Figure 1D), salivary gland, kidney, lung, pancreas, and prostate (Iber and Menshykau, 2013). Epithelial branching morphogenesis can involve lateral branching, planar bifurcation, orthogonal bifurcation, and (in some tissues) trifurcation of proliferating epithelial structures, which may be regulated by both local signaling gradients and physical constraints (Iber and Menshykau, 2013). The relative contribution of chemical and mechanical signaling, the interplay and integration of these environmental cues and the potential for pathway compensation, however, remains an area of active investigation. The continued development of genetically engineered mice with targeted disruptions to specific ligand-activated receptors and mechanosensing machinery will complement computational modeling of *in vivo* epithelial branching (Iber and Menshykau, 2013; Hannezo et al., 2017; Menshykau et al., 2019) and continue to shed new light on this important aspect of mammalian morphogenesis.

Once epithelial tissues are formed, they continue to experience mechanical load (Latorre et al., 2018). For example, mechanical forces prevent collapse of the lungs, which are considered a stress-supported structure (Waters et al., 2012). Moreover, epithelial cells in the lung undergo cyclical distension during the process of respiration, which can greatly vary in frequency and amplitude to precisely match physiological demands (Orr et al., 2006). Studies investigating the micromechanics of alveolar distension using real-time optical sectioning microscopy in rats have revealed that increasing the alveolar pressure from 5 cm H_2_O to 20 cm H_2_O (hyperinflation) increases alveolar diameter on average by 15% (Perlman and Bhattacharya, 2007). Expansion, however, is not uniform and alveolar type 1 cells experience greater load compared to type 2 cells (Perlman and Bhattacharya, 2007). This heterogeneity in the division of strain between neighboring cells is not uncommon in epithelial systems (Latorre et al., 2018). Like the lung, epithelial cells lining the urinary system (Nauli et al., 2003; Araki et al., 2008) as well as epithelial cells of functionally mature mammary gland (Stewart et al., 2019) and pancreas (Mamidi et al., 2018) experience a range of applied and intrinsic forces during normal function – including fluid flow, distension, compression, and contraction – which can both fluctuate and coincide. The magnitude, duration, and pulsatility of each of these forces is also likely to vary within and between tissues. Frictional forces exerted by flowing milk on luminal epithelial cells that line mammary ducts and alveoli, for example, conceivably change with milk composition and volume throughout the stages of lactation (Neville et al., 1991) and, owing to viscosity, are likely to generate distinct mechanical responses when compared to frictional forces exerted on cells lining other fluid transporting structures (Orr et al., 2006; Baeyens and Schwartz, 2016).

Multiple mechanisms exist to sense, transduce, and transmit force in epithelial cells and tissues, including mechanosensitive ion channels (e.g., transient receptor potential channels, the epithelial sodium channel, and PIEZO channels), focal adhesions and cadherin based cell-cell adhesions (Martinac, 2014; Gomez et al., 2011). It is important to note that these modes of force detection are not mutually exclusive and have been demonstrated to interact (McHugh et al., 2010; Nourse and Pathak, 2017). For example, in endothelial cells, shear force activates calcium entry though PIEZO1 channels, which promotes the proteolytic cleavage of cytoskeletal and focal adhesion proteins (Li et al., 2014).

Due to space constraints, we will limit our discussion in this mini-review to established and emerging roles of the recently identified, mechanically activated ion channel PIEZO1 in the development and physiology of mammalian epithelia. Roles for PIEZO1 in endothelial cells have recently been reviewed (Beech, 2018; Hyman et al., 2017) and will not be discussed further here.

## The Mechanosensitive Ion Channel Piezo1

PIEZO1 represents one half of the recently identified PIEZO family of mechanically activated ion channels (Coste et al., 2010). Since their discovery, important roles for both PIEZO1 and PIEZO2 have been described in mammals, with major insights gained from the development of conditional gene deletion models (Li et al., 2014; Ranade et al., 2014; Nonomura et al., 2018; Romac et al., 2018; Segel et al., 2019; Solis et al., 2019) and descriptions of hereditary mutations in humans (Zarychanski et al., 2012; Alper, 2017). These include the perception of touch (Murthy et al., 2018; Zhang et al., 2019), red blood cell volume regulation (Zarychanski et al., 2012; Cahalan et al., 2015) and vascular development (Li et al., 2014; Ranade et al., 2014). While both PIEZO1 and PIEZO2 are able to convert physical force into biochemical information, PIEZO2 is largely expressed in cells of neuronal origin, has recently been reviewed in this context (Anderson et al., 2017) and will thus not be discussed further here.

PIEZO1 was discovered in 2010 (Coste et al., 2010). Details of the structure and gating of this channel have only recently started to emerge and have largely been based on studies of mouse PIEZO1 (Ge et al., 2015; Saotome et al., 2018; Wang et al., 2018; Zhao et al., 2018; Lin et al., 2019). While investigations into the specific mechanisms of PIEZO1 gating are ongoing, studies using heterologous expression systems and reconstituted lipid bilayers have confirmed mechanical force as a direct regulator of channel opening (Syeda et al., 2016). A chemical activator of PIEZO1 has also been developed (Syeda et al., 2015), suggesting a complexity in channel gating that is yet to be fully understood. Upon its initial discovery, elevated *Piezo1* transcripts were identified in various mouse epithelial tissues, including bladder, colon, kidney, lung, and skin (Coste et al., 2010). Since then, a number of studies have investigated the physiological roles of PIEZO1 in specific epithelial tissues known to be the subject of mechanical forces (Peyronnet et al., 2013; Miyamoto et al., 2014; Martins et al., 2016; Michishita et al., 2016; Ihara et al., 2018; Romac et al., 2018; Stewart et al., 2019).

Roles for PIEZO1 channels in the morphogenesis and maintenance of epithelial tissues can also be inferred from studies linking this channel to life and death symmetry in healthy epithelia, including human colon epithelia (Eisenhoffer et al., 2012; Gudipaty et al., 2017). These studies have shown that overcrowding-strain in Madin-Darby canine kidney (MDCK) epithelial cell cultures resulted in live cell extrusions, which were blocked by inhibitors of Rho kinase and sphingosine-1 phosphate signaling as well as the non-selective ion channel blocker gadolinium (Gd^3+^) (Eisenhoffer et al., 2012). Genetic knockdown of *Piezo1* also inhibited homeostatic cell extrusions in the epidermis of developing zebrafish, producing localized epidermal cell masses (Eisenhoffer et al., 2012). This work set the scene for subsequent studies investigating cell extrusion and stretch in the development and homeostasis of other epithelial tissues *in vivo*, including the mammary gland (discussed in further detail below). This same group subsequently demonstrated that epithelial cell stretch at areas of low cell density was also detected and translated by PIEZO1, leading to cell division (Gudipaty et al., 2017). Comprehensive assessment of roles for PIEZO1 in controlled cell death and division in the developing mammalian embryo, however, have been hindered by embryonic lethality in *Piezo1-null* mice by E14.5, an effect that has largely been attributed to impaired vascular development (Li et al., 2014; Ranade et al., 2014). Analysis of epithelial development and branching morphogenesis using conditional *Piezo1* knockout mice will yield important new insights into the role of this channel in the formation of mammalian epithelial tissues.

The following sections of this review will specifically describe physiological roles for PIEZO1 mechanically activated ion channels in three epithelial organ-systems in mammals: the mammary gland, pancreas, and urinary system.

## Mammary Gland

As discussed, mammary epithelial cells experience mechanical forces in the embryo and during pubertal ductal morphogenesis. However, epithelial cells in the mammary gland also experience applied and cell-generated forces during lactation – when the mammary gland produces and expels milk to fulfill its sole biological role – and in the phase of post-lactational regression (involution) (Watson and Khaled, 2008; VanHouten et al., 2010; Davis et al., 2015). Shed cells have long been observed in the early phase of mammary gland involution (Watson, 2006). During this period, lactation can be resumed upon re-initiation of infant suckling (Watson and Kreuzaler, 2011). This is an important feature of mammalian biology, as it allows female mammals to continue nursing their offspring after modest periods of absence (e.g., during hunting or foraging). During this time, mammary epithelial cells experience substantial pressure due to milk accumulation in the alveolar lumen (Stewart et al., 2019). The apical extrusion of cells from the alveolar epithelium may help to preserve epithelial barrier integrity during this period of protracted tissue strain (Eisenhoffer et al., 2012). If it is live cells that are extruded from the epithelium during mammary gland involution to subsequently die via anoikis in the alveolar lumen as previously reported (Watson, 2006), this process may be mediated wholly or in-part by PIEZO1 (Eisenhoffer et al., 2012). To investigate this hypothesis, we recently generated mice with conditional deletion of *Piezo1* in luminal (secretory) mammary epithelial cells (Stewart et al., 2019, pre-print). The number of cleaved caspase 3 (CC3)-positive shed cells during the early phase of involution was not affected in mice with luminal cell *Piezo1* deletion (Stewart et al., 2019). In contrast to previous reports, however, CC3-positive cells were observed in both the alveolar lumen and the alveolar wall in this study, suggesting that, unlike live cell extrusions in other epithelial systems, cell death may precede cell extrusion in the involuting mouse mammary gland (Stewart et al., 2019), making a role for PIEZO1 unlikely.

Milk is ejected upon infant suckling, a response that most likely involves mechanical activation of sensory neurons at the nipple. This results in the release of centrally produced oxytocin into the circulation (Gimpl and Fahrenholz, 2001). Studies performing multi-scale activity imaging on live mammary tissue during lactation have revealed in unprecedented detail oxytocin-mediated, calcium-dependent contractions of alveolar myoepithelial cells (Stevenson et al., 2019, pre-print). These contractions cause alveolar units to eject their supply of milk and ductal myoepithelial contractions help to propel milk toward the nipple (Stevenson et al., 2019). These actomyosin-based contractions cause considerable warping of the ductal and alveolar structures, which may activate mechanosensing proteins. *Piezo1* deletion in luminal epithelial cells, however, has no effect on milk ejection or postnatal pup development (Stewart et al., 2019). These results, again, suggest that PIEZO1 channels either do not have a major role in the physiology of the functionally mature mammary gland or that their role can be compensated for by another pathway. It is interesting to note, however, that whilst suckling of one nipple generates a systemic oxytocin response, milk ejection is principally limited to the physically stimulated nipple. How a localized mechanical stimulus produces systemic release of a ligand to ultimately result in a highly localized response remains an unanswered question in mammalian biology. In any case, milk production, secretion, and coordinated ejection does not appear to depend on luminal cell expression of the mechanically activated ion channel *Piezo1* (Stewart et al., 2019).

## Pancreas

Pancreatic development commences at E9.5 in the mouse, with obvious branching morphogenesis of epithelial buds observed at around E12.5 (Villasenor et al., 2010). As mentioned above, embryonic organogenesis is a dynamic process involving significant cellular rearrangements and cell-cell and cell-extracellular matrix (ECM) interactions (Orr et al., 2006). Using a *Pdx1-Cre;Itgb1^*fl/fl*^* knockout mouse model, initiation of branching morphogenesis was shown to rely on β1 integrin-mediated interactions between pancreatic progenitors and localized regions of the ECM (Shih et al., 2016). Further supporting a role for mechanosignaling in pancreas development, are recent findings from Mamidi et al. (2018), who, using embryonic stem cells, identified a distinct role for ECM-integrin α5 interactions in driving ductal lineage specification of bipotent pancreatic progenitors. In addition to cell fate determination, integrin signaling regulates a range of cellular processes important in development, including remodeling of the cytoskeleton, cell adhesion, and migration (reviewed in Bökel and Brown, 2002). Early studies identified a role for *Piezo1* (known as *Fam38A* at the time) in regulating β1 integrin activation and cell adhesion in HeLa cells (McHugh et al., 2010). Since then, the majority of studies of PIEZO1 function have been assessed in the context of externally generated forces; however, recent findings by Pathak and colleagues, using super resolution imaging techniques, identified highly localized PIEZO1-dependent calcium flickers in response to cell generated traction forces (Ellefsen et al., 2019). It would be informative to investigate the impact of targeted *Piezo1* deletion under native conditions and to assess its role in epithelial cell-ECM signaling events in early embryonic development [for a comprehensive review of the relationship between PIEZO1, internally generated forces and the cytoskeleton, see (Nourse and Pathak, 2017)].

The mature exocrine pancreas consists of two main epithelial cell types: pancreatic ductal epithelial cells and acinar cells, with acinar cells representing the predominant cell type (>90%) (Romac et al., 2018; Gál et al., 2019). Unique to the pancreas, is its exquisite sensitivity to touch (Romac et al., 2018). Recently, *Piezo1* expression was confirmed in pancreatic acinar cells, where it has been proposed to mediate touch and pressure sensitivity (Romac et al., 2018). *In vivo* studies performed by Romac et al. (2018) showed that mouse pancreas exposed to the PIEZO1 agonist, Yoda1, exhibited signs of cellular injury similar to that caused by high intraductal pressure. Furthermore, damage caused by high intraductal pressure is reduced in the presence of GsMTx4 as well as by targeted deletion of *Piezo1* in acinar cells (Romac et al., 2018). While levels of *Piezo1* mRNA specifically in pancreatic ductal epithelial cells have not been reported, perfusion of mouse pancreas tissue slices with the bile acid, chenodeoxycholic acid, is associated with transient calcium increases in these cells (Gál et al., 2019). Given its role in the transport of pancreatic secretions and this tissue’s sensitivity to physical obstruction and compression (Hegyi and Petersen, 2013; Romac et al., 2018), it would be valuable to know whether PIEZO1 is also active in epithelial cells lining the pancreatic duct.

To date, few studies have assessed the role of PIEZO1 in the pancreas. As mentioned above, the cell-intrinsic forces encountered during its development, combined with its unique pressure sensitivity, makes the pancreas an interesting candidate for future investigations into how the magnitude and nature of mechanical stimulation can influence its normal growth and functioning.

## Urinary System

Unlike the mammary gland and pancreas, epithelial cells of the urinary system have been the focus of a number of studies investigating physiological roles of PIEZO1 (Peyronnet et al., 2013; Miyamoto et al., 2014; Martins et al., 2016; Michishita et al., 2016; Ihara et al., 2018). Fluid flow, distension, and contraction are just some examples of the dynamic forces faced by epithelial cells lining the urinary system, which includes the urinary bladder, ureters, and renal pelvis (Andersson and Arner, 2004). Of the epithelial tissues assessed by Coste et al. (2010) mouse *Piezo1* transcript levels were shown to be highly expressed in the bladder, supporting a potential role in mechanosensory processes in tissues of the urinary system. Indeed, an early study assessing PIEZO1 function in cultured proximal convoluted tubule (PCT) cells identified a reduction in endogenous stretch activated channel activity upon siRNA-mediated *Piezo1* knockdown (Peyronnet et al., 2013). Further, this group showed that stretch activated channel activity is reduced upon co-expression of the calcium permeable ion channel, Polycystin-2 (PC2), and *Piezo1*, suggesting a regulatory role of PC2 (Peyronnet et al., 2013). The physiological relevance of this interaction remains unknown; however, it would be interesting to evaluate the expression and activity of PIEZO1 in disease states in which PC2 is mutated, such as autosomal dominant polycystic kidney disease (Mochizuki et al., 1996).

The urothelium, a highly specialized epithelium lining the urinary bladder, ureters, and renal pelvis, plays an important role in sensing and responding to forces generated within the bladder (Mochizuki et al., 2009; Birder, 2011; Negoro et al., 2014). Similar to studies in PCT cells, knockdown of *Piezo1* in primary cultured mouse bladder urothelial cells resulted in a reduced response to mechanical stimuli (Miyamoto et al., 2014). This group also showed a reduction in urothelial cell stretch-mediated adenosine triphosphate (ATP) release as a consequence of either *Piezo1* knockdown or GsMTx4 treatment, illustrating a potential role for PIEZO1 channels in the translation of mechanical force into downstream signaling events in bladder cells. Yet another function of PIEZO1 in epithelia of the urinary tract was recently identified, this time in collecting duct epithelial cells of the kidney (Martins et al., 2016). Using a conditional *Piezo1* knockout model targeted to epithelial cells of the collecting ducts and renal tubules in adult mice, Martins et al. (2016) demonstrated a role for PIEZO1 in the regulation of urine osmolarity post-dehydration or fasting, via an as yet unknown mechanism.

As discussed briefly in the studies presented above, there is mounting evidence for a role for PIEZO1 in the normal functioning of various epithelial cell types of the urinary tract. Currently, the majority of studies assessing PIEZO1 have largely relied on the use of *in vitro* primary and immortalized cell lines. It will be interesting to see whether these phenotypes are reproduced and expounded in the future using targeted knockout animal models.

## Concluding Remarks

In this review, we considered the physical forces that are exerted on epithelial cells as they evolve into complex tissues and perform their basic physiological functions. Applied and cell-generated forces are encountered in a developmental stage- and tissue-specific manner as cells proliferate, reorganize and interact with neighboring cells and their physical environment (Chen, 2008; Latorre et al., 2018). The sheer scale of force experienced (often simultaneously) by cells in multicellular systems is unlikely to be detected, decoded, and transferred by a single protein or family of proteins (Orr et al., 2006), nor do physical forces act in isolation from chemical signaling (Orr et al., 2006; Li et al., 2018; Stewart and Davis, 2019). Whilst our focus in this mini-review has been on PIEZO1, it is important to note that PIEZO1 is only one recently identified piece of a highly complex puzzle. It is also important to note that mechanical tension is not only a feature of normal development and physiology, as discussed here, it is also an integral component of disease signaling. Asthma (Gunst and Tang, 2000; Waters et al., 2012), pulmonary fibrosis (Waters et al., 2012), pancreatitis (Romac et al., 2018), and breast cancer (Li et al., 2015) are all examples of epithelial pathologies that are linked to altered mechanosensing. Lessons learned from epithelial mechanobiology will no doubt provide important new insights into epithelial “mechanopathologies.”

## Ethics Statement

Animal experimentation was carried out in accordance with the Australian Code for the Care and Use of Animals for Scientific Purposes and the Queensland Animal Care and Protection Act (2001), with local animal ethics committee approval (The University of Queensland Health Sciences Animal Ethics Committee, The University of Queensland).

## Author Contributions

TS and FD contributed to the writing and editing of this manuscript.

## Conflict of Interest

The authors declare that the research was conducted in the absence of any commercial or financial relationships that could be construed as a potential conflict of interest.

## References

[B1] Alper S. L. (2017). Genetic diseases of PIEZO1 and PIEZO2 dysfunction. *Curr. Top. Membr.* 79 97–134. 10.1016/bs.ctm.2017.01.001 28728825

[B2] Anderson E. O. O. Schneider E. R. R. Bagriantsev S. N. N. (2017). Piezo2 in cutaneous and proprioceptive mechanotransduction in vertebrates. *Curr. Top. Membr.* 79 197–217. 10.1016/bs.ctm.2016.11.002 28728817 PMC5630267

[B3] Andersson K.-E. Arner A. (2004). Urinary bladder contraction and relaxation: physiology and pathophysiology. *Physiol. Rev.* 84 935–986. 10.1152/physrev.00038.2003 15269341

[B4] Araki I. Du S. Kobayashi H. Sawada N. Mochizuki T. Zakoji H. (2008). Roles of mechanosensitive ion channels in bladder sensory transduction and overactive bladder. *Int. J. Urol.* 15 681–687. 10.1111/j.1442-2042.2008.02052.x 18462357

[B5] Baeyens N. Schwartz M. A. (2016). Biomechanics of vascular mechanosensation and remodeling. *Mol. Biol. Cell* 27 7–11. 10.1091/mbc.E14-11-1522 26715421 PMC4694763

[B6] Beech D. J. (2018). Endothelial Piezo1 channels as sensors of exercise. *J. Physiol.* 596 979–984. 10.1113/JP274396 29194632 PMC5851887

[B7] Behrndt M. Salbreux G. Campinho P. Hauschild R. Oswald F. Roensch J. (2012). Forces driving epithelial spreading in zebrafish gastrulation. *Science* 338 257–260. 10.1126/science.1224143 23066079

[B8] Beloussov L. V. (1980). The role of tensile fields and contact cell polarization in the morphogenesis of amphibian axial rudiments. *Wilhelm Roux’s Arch. Dev. Biol.* 188 1–7. 10.1007/BF00848603 28305148

[B9] Birder L. A. (2011). “Urothelial signaling,” in *Urinary Tract. Handbook of Experimental Pharmacology*, Vol. 2011 eds Andersson K. E. Michel M. (Berlin: Springer).

[B10] Bökel C. Brown N. H. (2002). Integrins in development: moving on, responding to, and sticking to the extracellular matrix. *Dev. Cell.* 3 311–321. 12361595 10.1016/s1534-5807(02)00265-4

[B11] Cahalan S. M. Lukacs V. Ranade S. S. Chien S. Bandell M. Patapoutian A. (2015). Piezo1 links mechanical forces to red blood cell volume. *eLife* 4:e07370. 10.7554/eLife.07370 26001274 PMC4456639

[B12] Chen C. S. (2008). Mechanotransduction – a field pulling together? *J. Cell Sci.* 121 3285–3292. 10.1242/jcs.023507 18843115

[B13] Coste B. Mathur J. Schmidt M. Earley T. J. Ranade S. Petrus M. J. (2010). Piezo1 and Piezo2 are essential components of distinct mechanically activated cation channels. *Science* 330 55–60. 10.1126/science.1193270 20813920 PMC3062430

[B14] Cowin P. Wysolmerski J. (2010). Molecular mechanisms guiding embryonic mammary gland development. *Cold Spring Harb. Perspect. Biol.* 2:a003251. 10.1101/cshperspect.a003251 20484386 PMC2869520

[B15] Davis F. M. Janoshazi A. Janardhan K. S. Steinckwich N. D’Agostin D. M. Petranka J. G. (2015). Essential role of Orai1 store-operated calcium channels in lactation. *Proc. Natl. Acad. Sci. U.S.A.* 112 5827–5832. 10.1073/pnas.1502264112 25902527 PMC4426473

[B16] Dunbar M. E. Dann P. R. Zhang J.-P. Wysolmerski J. J. Robinson G. W. Hennighausen L. (1999). Parathyroid hormone-related protein signaling is necessary for sexual dimorphism during embryonic mammary development. *Development* 126 3485–3493. 10409496 10.1242/dev.126.16.3485

[B17] Eisenhoffer G. T. Loftus P. D. Yoshigi M. Otsuna H. Chien C. B. Morcos P. A. (2012). Crowding induces live cell extrusion to maintain homeostatic cell numbers in epithelia. *Nature* 484 546–549. 10.1038/nature10999 22504183 PMC4593481

[B18] Ellefsen K. L. Holt J. R. Chang A. C. Nourse J. L. Arulmoli J. Mekhdjian A. H. (2019). Myosin-II mediated traction forces evoke localized Piezo1-dependent Ca2+ flickers. *Commun. Biol.* 2:298. 10.1038/s42003-019-0514-3 31396578 PMC6685976

[B19] Gál E. Dolenšek J. Stožer A. Pohorec V. Ébert A. Venglovecz V. (2019). A novel in situ approach to studying pancreatic ducts in mice. *Front. Physiol.* 10:938. 10.3389/fphys.2019.00938 31396104 PMC6668154

[B20] Ge J. Li W. Zhao Q. Li N. Chen M. Zhi P. (2015). Architecture of the mammalian mechanosensitive Piezo1 channel. *Nature* 527 64–69. 10.1038/nature15247 26390154

[B21] Gimpl G. Fahrenholz F. (2001). The oxytocin receptor system: structure, function, and regulation. *Physiol. Rev.* 81 629–683. 10.1152/physrev.2001.81.2.629 11274341

[B22] Gomez G. A. McLachlan R. W. Yap A. S. (2011). Productive tension: force-sensing and homeostasis of cell-cell junctions. *Trends Cell Biol.* 21 499–505. 10.1016/j.tcb.2011.05.006 21763139

[B23] Gudipaty S. A. Lindblom J. Loftus P. D. Redd M. J. Edes K. Davey C. F. (2017). Mechanical stretch triggers rapid epithelial cell division through Piezo1. *Nature* 543 118–121. 10.1038/nature21407 28199303 PMC5334365

[B24] Gunst S. J. Tang D. D. (2000). The contractile apparatus and mechanical properties of airway smooth muscle. *Eur. Respir. J.* 15 600–616. 10.1034/j.1399-3003.2000.15.29.x 10759460

[B25] Guo C. L. Ouyang M. Yu J. Y. Maslov J. Price A. Shen C. Y. (2012). Long-range mechanical force enables self-assembly of epithelial tubular patterns. *Proc. Natl. Acad. Sci. U.S.A.* 109 5576–5582. 10.1073/pnas.1114781109 22427356 PMC3326479

[B26] Hannezo E. Scheele C. L. G. J. Moad M. Drogo N. Heer R. Sampogna R. V. (2017). A unifying theory of branching morphogenesis. *Cell* 171 242–255. 10.1016/j.cell.2017.08.026 28938116 PMC5610190

[B27] Hegyi P. Petersen O. H. (2013). The exocrine pancreas: the acinar-ductal tango in physiology and pathophysiology. *Rev. Physiol. Biochem. Pharmacol.* 165 1–30. 10.1007/112_2013_14 23881310

[B28] Heuberger B. Fitzka I. Wasner G. Kratochwil K. (2006). Induction of androgen receptor formation by epithelium-mesenchyme interaction in embryonic mouse mammary gland. *Proc. Natl. Acad. Sci. U.S.A.* 79 2957–2961. 10.1073/pnas.79.9.2957 6953441 PMC346327

[B29] Hyman A. J. Tumova S. Beech D. J. (2017). Piezo1 channels in vascular development and the sensing of shear stress. *Curr. Top. Membr.* 79 37–57. 10.1016/bs.ctm.2016.11.001 28728823

[B30] Iber D. Menshykau D. (2013). The control of branching morphogenesis. *Open Biol.* 3:130088. 10.1098/rsob.130088 24004663 PMC3787747

[B31] Ihara T. Mitsui T. Nakamura Y. Kanda M. Tsuchiya S. Kira S. (2018). The oscillation of intracellular Ca2+ influx associated with the circadian expression of Piezo1 and TRPV4 in the bladder urothelium. *Sci. Rep.* 8:5699. 10.1038/s41598-018-23115-w 29632308 PMC5890282

[B32] Keller R. Davidson L. A. Shook D. R. (2003). How we are shaped: the biomechanics of gastrulation. *Differentiation* 71 171–205. 10.1046/j.1432-0436.2003.710301.x 12694202

[B33] Krieg M. Arboleda-Estudillo Y. Puech P. H. Käfer J. Graner F. Müller D. J. (2008). Tensile forces govern germ-layer organization in zebrafish. *Nat. Cell Biol.* 10 429–436. 10.1038/ncb1705 18364700

[B34] Latorre E. Kale S. Casares L. Gómez-González M. Uroz M. Valon L. (2018). Active superelasticity in three-dimensional epithelia of controlled shape. *Nature* 563 203–208. 10.1038/s41586-018-0671-4 30401836 PMC6520229

[B35] Lewis W. H. (1947). Mechanics of invagination. *Anat. Rec.* 97 139–156. 10.1002/ar.1090970203 20284907

[B36] Li C. Rezania S. Kammerer S. Sokolowski A. Devaney T. Gorischek A. (2015). Piezo1 forms mechanosensitive ion channels in the human MCF-7 breast cancer cell line. *Sci. Rep.* 5:8364. 10.1038/srep08364 25666479 PMC4322926

[B37] Li J. Hou B. Tumova S. Muraki K. Bruns A. Ludlow M. J. (2014). Piezo1 integration of vascular architecture with physiological force. *Nature* 515 279–282. 10.1038/nature13701 25119035 PMC4230887

[B38] Li J. Wang Z. Chu Q. Jiang K. Li J. Tang N. (2018). The strength of mechanical forces determines the differentiation of alveolar epithelial cells. *Dev. Cell* 44 297–312. 10.1016/j.devcel.2018.01.008 29408236

[B39] Lin Y. C. Guo Y. R. Miyagi A. Levring J. MacKinnon R. Scheuring S. (2019). Force-induced conformational changes in PIEZO1. *Nature* 573 230–234. 10.1038/s41586-019-1499-2 31435018 PMC7258172

[B40] Mamidi A. Prawiro C. Seymour P. A. de Lichtenberg K. H. Jackson A. Serup P. (2018). Mechanosignalling via integrins directs fate decisions of pancreatic progenitors. *Nature* 564 114–118. 10.1038/s41586-018-0762-2 30487608

[B41] Martinac B. (2014). The ion channels to cytoskeleton connection as potential mechanism of mechanosensitivity. *Biochim. Biophys. Acta Biomembr.* 1838 682–691. 10.1016/j.bbamem.2013.07.015 23886913

[B42] Martins J. R. Penton D. Peyronnet R. Arhatte M. Moro C. Picard N. (2016). Piezo1-dependent regulation of urinary osmolarity. *Pflugers Arch. Eur. J. Physiol.* 468 1197–1206. 10.1007/s00424-016-1811-z 27023350

[B43] McDole K. Guignard L. Amat F. Berger A. Malandain G. Royer L. A. (2018). In toto imaging and reconstruction of post-implantation mouse development at the single-cell level. *Cell* 175 859–876. 10.1016/j.cell.2018.09.031 30318151

[B44] McHugh B. J. Buttery R. Lad Y. Banks S. Haslett C. Sethi T. (2010). Integrin activation by Fam38A uses a novel mechanism of R-Ras targeting to the endoplasmic reticulum. *J. Cell Sci.* 123 51–61. 10.1242/jcs.056424 20016066 PMC2794710

[B45] Menshykau D. Michos O. Lang C. Conrad L. McMahon A. P. Iber D. (2019). Image-based modeling of kidney branching morphogenesis reveals GDNF-RET based Turing-type mechanism and pattern-modulating WNT11 feedback. *Nat. Commun.* 10:239. 10.1038/s41467-018-08212-8 30651543 PMC6484223

[B46] Michishita M. Yano K. Tomita K. I. Matsuzaki O. Kasahara K. I. (2016). Piezo1 expression increases in rat bladder after partial bladder outlet obstruction. *Life Sci.* 166 1–7. 10.1016/j.lfs.2016.10.017 27756599

[B47] Miller C. J. Davidson L. A. (2013). The interplay between cell signalling and mechanics in developmental processes. *Nat. Rev. Genet.* 14 733–744. 10.1038/nrg3513 24045690 PMC4056017

[B48] Miyamoto T. Mochizuki T. Nakagomi H. Kira S. Watanabe M. Takayama Y. (2014). Functional role for Piezo1 in stretch-evoked Ca2+ influx and ATP release in Urothelial cell cultures. *J. Biol. Chem.* 289 16565–16575. 10.1074/jbc.M113.528638 24759099 PMC4047422

[B49] Mochizuki T. Sokabe T. Araki I. Fujishita K. Shibasaki K. Uchida K. (2009). The TRPV4 cation channel mediates stretch-evoked Ca2+ influx and ATP release in primary urothelial cell cultures. *J. Biol. Chem.* 284 21257–21264. 10.1074/jbc.M109.020206 19531473 PMC2755849

[B50] Mochizuki T. Wu G. Hayashi T. Xenophontos S. L. Veldhuisen B. Saris J. J. (1996). PKD2, a gene for polycystic kidney disease that encodes an integral membrane protein. *Science* 272 1339–1342. 10.1126/science.272.5266.1339 8650545

[B51] Murthy S. E. Loud M. C. Daou I. Marshall K. L. Schwaller F. Kühnemund J. (2018). The mechanosensitive ion channel Piezo2 mediates sensitivity to mechanical pain in mice. *Sci. Transl. Med.* 10:eaat9897. 10.1126/scitranslmed.aat9897 30305457 PMC6709986

[B52] Nauli S. M. Alenghat F. J. Luo Y. Williams E. Vassilev P. Li X. (2003). Polycystins 1 and 2 mediate mechanosensation in the primary cilium of kidney cells. *Nat. Genet.* 33 129–137. 10.1038/ng1076 12514735

[B53] Negoro H. Urban-Maldonado M. Liou L. S. Spray D. C. Thi M. M. Suadicani S. O. (2014). Pannexin 1 channels play essential roles in urothelial mechanotransduction and intercellular signaling. *PLoS One* 9:e106269. 10.1371/journal.pone.0106269 25170954 PMC4149561

[B54] Nelson C. M. Jean R. P. Tan J. L. Liu W. F. Sniadecki N. J. Spector A. A. (2005). Emergent patterns of growth controlled by multicellular form and mechanics. *Proc. Natl. Acad. Sci. U.S.A.* 102 11594–11599. 10.1073/pnas.0502575102 16049098 PMC1187971

[B55] Neville M. C. Allen J. C. Archer P. C. Casey C. E. Seacat J. Keller R. P. (1991). Studies in human lactation: milk volume and nutrient composition during weaning and lactogenesis. *Am. J. Clin. Nutr.* 54 81–92. 10.1093/ajcn/54.1.81 2058592

[B56] Nonomura K. Lukacs V. Sweet D. Goddard L. Kanie A. Whitwam T. (2018). Mechanically activated ion channel PIEZO1 is required for lymphatic valve formation. *Proc. Natl. Acad. Sci. U.S.A.* 115 12817–12822. 10.1073/pnas.1817070115 30482854 PMC6294938

[B57] Nourse J. L. Pathak M. M. (2017). How cells channel their stress: interplay between Piezo1 and the cytoskeleton. *Semin. Cell Dev. Biol.* 71 3–12. 10.1016/j.semcdb.2017.06.018 28676421 PMC6070642

[B58] Orr A. W. Helmke B. P. Blackman B. R. Schwartz M. A. (2006). Mechanisms of mechanotransduction. *Dev. Cell* 10 11–20. 16399074 10.1016/j.devcel.2005.12.006

[B59] Perlman C. E. Bhattacharya J. (2007). Alveolar expansion imaged by optical sectioning microscopy. *J. Appl. Physiol.* 103 1037–1044. 10.1152/japplphysiol.00160.2007 17585045

[B60] Peyronnet R. Martins J. R. Duprat F. Demolombe S. Arhatte M. Jodar M. (2013). Piezo1-dependent stretch-activated channels are inhibited by Polycystin-2 in renal tubular epithelial cells. *EMBO Rep.* 14 1143–1148. 10.1038/embor.2013.170 24157948 PMC3981085

[B61] Ranade S. S. Qiu Z. Woo S. H. Hur S. S. Murthy S. E. Cahalan S. M. (2014). Piezo1, a mechanically activated ion channel, is required for vascular development in mice. *Proc. Natl. Acad. Sci. U.S.A.* 111 10347–10352. 10.1073/pnas.1409233111 24958852 PMC4104881

[B62] Romac J. M. J. Shahid R. A. Swain S. M. Vigna S. R. Liddle R. A. (2018). Piezo1 is a mechanically activated ion channel and mediates pressure induced pancreatitis. *Nat. Commun.* 9:1715. 10.1038/s41467-018-04194-9 29712913 PMC5928090

[B63] Saotome K. Murthy S. E. Kefauver J. M. Whitwam T. Patapoutian A. Ward A. B. (2018). Structure of the mechanically activated ion channel Piezo1. *Nature* 554 481–486. 10.1038/nature25453 29261642 PMC6010196

[B64] Segel M. Neumann B. Hill M. F. E. Weber I. P. Viscomi C. Zhao C. (2019). Niche stiffness underlies the ageing of central nervous system progenitor cells. *Nature* 573 130–134. 10.1038/s41586-019-1484-9 31413369 PMC7025879

[B65] Shih H. P. Panlasigui D. Cirulli V. Sander M. (2016). ECM signaling regulates collective cellular dynamics to control pancreas branching morphogenesis. *Cell Rep.* 14 169–179. 10.1016/j.celrep.2015.12.027 26748698 PMC4715768

[B66] Solis A. G. Bielecki P. Steach H. R. Sharma L. Harman C. C. D. Yun S. (2019). Mechanosensation of cyclical force by PIEZO1 is essential for innate immunity. *Nature* 573 69–74. 10.1038/s41586-019-1485-8 31435009 PMC6939392

[B67] Stevenson A. J. Vanwalleghem G. Stewart T. A. Condon N. D. Lloyd-Lewis B. Marino N. (2019). Multiscale activity imaging in the mammary gland reveals how oxytocin enables lactation. *bioRxiv* [Preprint].

[B68] Stewart T. A. Davis F. M. (2019). An element for development: calcium signaling in mammalian reproduction and development. *Biochim. Biophys. Acta Mol. Cell Res.* 1866 1230–1238. 10.1016/j.bbamcr.2019.02.016 30826333

[B69] Stewart T. A. Hughes K. Stevenson A. S. J. Marino N. Ju A. J. L. Morehead M. (2019). Mammary mechanobiology: mechanically-activated ion channels in lactation and involution. *bioRxiv* [Preprint]. 10.1242/jcs.248849 33262312

[B70] Syeda R. Florendo M. N. Cox C. D. Kefauver J. M. Santos J. S. Martinac B. (2016). Piezo1 channels are inherently mechanosensitive. *Cell Rep.* 17 1739–1746. 10.1016/j.celrep.2016.10.033 27829145 PMC5129625

[B71] Syeda R. Xu J. Dubin A. E. Coste B. Mathur J. Huynh T. (2015). Chemical activation of the mechanotransduction channel piezo1. *eLife* 4:e07369. 10.7554/eLife.07369 PMC4456433 26001275

[B72] VanHouten J. Sullivan C. Bazinet C. Ryoo T. Camp R. Rimm D. L. (2010). PMCA2 regulates apoptosis during mammary gland involution and predicts outcome in breast cancer. *Proc. Natl. Acad. Sci. U.S.A.* 107 11405–11410. 10.1073/pnas.0911186107 20534448 PMC2895115

[B73] Villasenor A. Chong D. C. Henkemeyer M. Cleaver O. (2010). Epithelial dynamics of pancreatic branching morphogenesis. *Development* 137 4295–4305. 10.1242/dev.052993 21098570 PMC2990215

[B74] Wang Y. Chi S. Guo H. Li G. Wang L. Zhao Q. (2018). A lever-like transduction pathway for long-distance chemical- and mechano-gating of the mechanosensitive Piezo1 channel. *Nat. Commun.* 9:1300. 10.1038/s41467-018-03570-9 29610524 PMC5880808

[B75] Waters C. M. Roan E. Navajas D. (2012). Mechanobiology in lung epithelial cells: measurements, perturbations, and responses. *Compr. Physiol.* 2 1–29. 10.1002/cphy.c100090 23728969 PMC4457445

[B76] Watson C. J. (2006). Key stages in mammary gland development Involution: apoptosis and tissue remodelling that convert the mammary gland from milk factory to a quiescent organ. *Breast Cancer Res.* 8:203. 10.1186/bcr1401 PMC1557708 16677411

[B77] Watson C. J. Khaled W. T. (2008). Mammary development in the embryo and adult: a journey of morphogenesis and commitment. *Development* 135 995–1003. 10.1242/dev.005439 18296651

[B78] Watson C. J. Kreuzaler P. A. (2011). Remodeling mechanisms of the mammary gland during involution. *Int. J. Dev. Biol.* 55 757–762. 10.1387/ijdb.113414cw 22161832

[B79] Zarychanski R. Schulz V. P. Houston B. L. Maksimova Y. Houston D. S. Smith B. (2012). Mutations in the mechanotransduction protein PIEZO1 are associated with hereditary xerocytosis. *Blood* 120 1908–1915. 10.1182/blood-2012-04-422253 22529292 PMC3448561

[B80] Zhang M. Wang Y. Geng J. Zhou S. Xiao B. (2019). Mechanically activated piezo channels mediate touch and suppress acute mechanical pain response in mice. *Cell Rep.* 26 1419.e4–1431.e4. 10.1016/j.celrep.2019.01.056 30726728

[B81] Zhao Q. Zhou H. Chi S. Wang Y. Wang J. Geng J. (2018). Structure and mechanogating mechanism of the piezo1 channel. *Nature* 554 487–492. 10.1038/nature25743 29469092

